# Towards Wiser Prescribing of Antibiotics in Dental Practice: What Pharmacists Want Dentists to Know

**DOI:** 10.3390/dj12110345

**Published:** 2024-10-29

**Authors:** Abrar K. Thabit, Nourah M. Aljereb, Omnia M. Khojah, Hanan Shanab, Arwa Badahdah

**Affiliations:** 1 Department of Pharmacy Practice, Faculty of Pharmacy, King Abdulaziz University, Jeddah 22254-2265, Saudi Arabia; 2 Faculty of Pharmacy, King Abdulaziz University, Jeddah 22254-2265, Saudi Arabia; 3 Department of Oral and Maxillofacial Surgery and Diagnostic Sciences, College of Dentistry, Majmaah University, Majmaah 11952, Saudi Arabia; h.hamzih@mu.edu.sa; 4 Department of Periodontology, Faculty of Dentistry, King Abdulaziz University, Jeddah 22254-2265, Saudi Arabia

**Keywords:** antibiotics, dentistry, antibiotic misuse, antimicrobial stewardship, dental procedures, pharmacy, pharmacists, drug prescriptions

## Abstract

Antibiotics have various indications for treatment and prophylaxis in dental practice. While only a handful of antibiotics are typically prescribed in dentistry, unlike in medicine, clear indications and appropriate dosing and duration remain controversial among antibiotic-prescribing dentists, which may result in inappropriate or excessive antibiotic prescriptions. This practice can increase the risk of antibiotic resistance and expose patients to unnecessary side effects. Moreover, the concept of antibiotic stewardship in dental practice remains in its early stages. This review was developed by pharmacists (general and infectious disease pharmacists) in collaboration with a periodontist and a maxillofacial surgeon to provide an antibiotic prescription guide for dentists who frequently prescribe antibiotics. It also sheds light on antibiotic stewardship. The review discusses in detail antibiotic indications for treatment and prophylaxis in dental practice and provides tables that can be used by dentists in their everyday practice. It also discusses the concept of antibiotic stewardship and provides recommendations that can be applied to the practice of antibiotic prescribing in dentistry. Antibiotic prescribing in dental practice should be limited to cases with documented infections or when indicated for prophylaxis. Every dentist can act as an antibiotic steward by prescribing antibiotics wisely and only when necessary, using their discernment to identify appropriate cases and exclude those that do not meet infection criteria. Collaboration with pharmacists is encouraged to provide such recommendations and implement antibiotic stewardship interventions, such as developing antibiotic prescription protocols.

## 1. Introduction

Antibiotics play a critical role in dentistry. They help prevent severe health complications such as infective endocarditis (IE) in at-risk patients and treat infections resulting from oral diseases. Oral infections can adversely affect the soft and hard tissues of the oral cavity and, in rare cases, lead to life-threatening facial space involvement. Pain and swelling are common signs of oral infections, with fever occurring less frequently, typically in cases of systemic spread. As such, dentists prescribe antibiotics for therapeutic or prophylactic purposes as an adjunct to clinical intervention, but not as a substitute for definitive treatment. Antibiotics should be used judiciously for rational indications, considering the appropriate antibiotic, optimal dosage, and a suitable duration of therapy to avoid the consequences and risks associated with antibiotic misuse [1,2].

The risk of antibiotic misuse lies in its adverse effects and the emergence of antibiotic-resistant bacteria. Adverse effects may include gastrointestinal and hematological problems, as well as hypersensitivity reactions. Furthermore, disrupting the normal enteric flora can lead to opportunistic infections, such as *Clostridioides difficile* infections [3,4]. Antimicrobial resistance (AMR) is a major threat to public health worldwide. It results from the exposure of microorganisms to antibiotics, leading to the development of resistant strains that are no longer killed or inhibited by these antibiotics, thus resulting in the emergence of serious infections that can be difficult to treat. As such, antibiotic misuse contributes to the increase in AMR [5].

This review has two objectives: First, to summarize key information on antibiotics commonly used in dental practice to serve as a prescribing guide for dentists who frequently prescribe antibiotics. Second, to describe antibiotic stewardship and how it can be applied to antibiotic prescribing in dentistry. The review was developed by a multidisciplinary team, including pharmacists (general and infectious disease pharmacists) collaborating with a periodontist and a maxillofacial surgeon.

## 2. Overview of Oral Microorganisms and Infections

**Figure 1:**
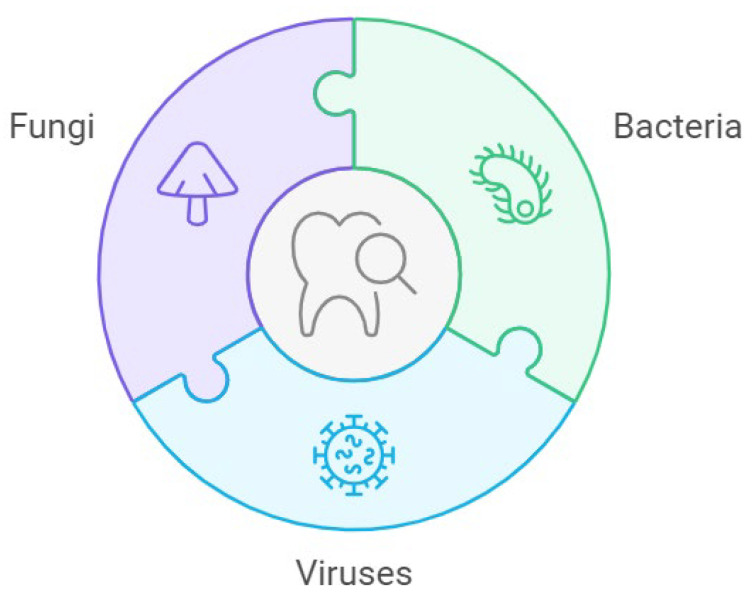
Components of the oral microbiome.

An abundant and diversified microbial population lives in the mouth because of the ongoing exposure of the oral cavity to the external environment (Figure 1). The Expanded Human Oral Microbiome Database, updated in 2017, shows that the oral environment is home to more than 772 different types of microorganisms (including bacteria, fungi, and protozoa) [6]. The development of the oral microbiome is significantly influenced by temperature, nutrition, pH, and feeding practices [7,8]. Maintaining stable microbial populations is essential for maintaining oral health, as diseases may develop when this equilibrium is disrupted, and more pathogenic species outgrow the commensals [8,9]. Different species of Gram-positive, Gram-negative, and anaerobic bacteria inhabit the oral cavity as normal flora, which are also implicated in oral and systemic infections, namely, the viridans group *Streptococci*, *Prevotella* spp., *Fusobacterium* spp., *Porphyromonas gingivalis*, and *Bacteroides* spp. [8,10,11].

Dental plaque is a biofilm composed primarily of microbial colonies bound to intra-oral hard surfaces and protected by an extracellular matrix. Dental plaque is the primary etiologic factor associated with all forms of oral diseases, including caries, periodontal diseases, and endodontic infections [12]. *Streptococcus mutans*, *Bifidobacteria*, and *Lactobacilli* are the most important species of acidogenic–aciduric bacteria currently recognized as the main etiologic factors of dental caries. Compared to the initiator *Streptococcus mutans*, both *Bifidobacteria* and *Lactobacilli* are greater promoters of the progression of dental caries, as acids promote the dissolving of the hard structure of the tooth [13]. *Propionobacterium* and *Scarvidia* are other identified bacterial species associated with the development of dental caries [7].

Periodontitis is a chronic, progressive inflammatory disease of the periodontium caused by specific microorganisms that result in the loss of the periodontal attachment of the host, with varying rates of disease progression. Clinically and radiographically, it is detected as clinical attachment loss (CAL), bone loss, increased probing depth, and gingival recession. The pathogenic component of periodontal disease has long been suggested to be the dental plaque biofilm, especially the subgingival composition, which is affected by the anaerobic condition and the availability of blood products in the subgingival environment. *Porphyromonas gingivalis*, *Aggregatibacter actinomycetemcomitans*, *Treponema denticola*, and *Tannerella forsythia* are considered the most pathogenic species in the onset and progression of periodontitis. The microbial–host interaction is crucial in the development of the disease [14]. The course of the disease is determined by the immune response to the bacterial challenge, which is not an isolated event; instead, it is influenced by various host and environmental factors. Smoking and diabetes are two risk factors that increase one’s vulnerability to periodontal disease [15,16]. Furthermore, periodontitis may influence the progression of or increase the risk of certain systemic diseases. The effects of periodontal infection have been linked to several conditions, including diabetes mellitus, coronary heart disease, and preterm birth [17].

Endodontic infections result from the bacterial invasion of the dental pulp with species primarily associated with carious lesions. Common species include dark-pigmented bacteria, *Peptostreptococcus, Eubacterium, Fusobacterium* spp., *Prevotella* spp., *Porphyromonas* spp., *Tannerella forsythia*, *Dialister* spp., *Treponema* spp., and *Actinomyces* spp. [18,19]. Moreover, *Enterococcus faecalis* is a facultative anaerobe that can survive in the poor nutritional conditions in root-filled teeth; thus, it is frequently detected in cases of failed root canal therapy [20]. In less common situations, infections of odontogenic origin are not confined only to the oral cavity but can also spread to facial spaces, causing facial space infection, which could be a life-threatening condition.

Infections caused by oral microorganisms are not confined solely to the oral cavity or the surrounding facial tissues. The access of oral microorganisms to the bloodstream after invasive dental procedures can result in bacteremia, in which vulnerable patients may develop IE. Gram-positive coccis (*Streptococci*, *Staphylococci*, and *Enterococci*) account for 80–90% of IE cases. In the developed world, *Staphylococcus aureus* is responsible for 30% of such infections, whereas the more serious fungal endocarditis accounts for only about 1% of all cases [21].

## 3. Indications for Therapeutic Antibiotics or Prevention of Postoperative Infections

Several dental conditions and procedures require antibiotics to manage active infections or improve treatment outcomes. Detailed below are descriptions of common indications for antibiotics used in dental practice. Overall, the most commonly prescribed antibiotics are penicillins, such as amoxicillin alone or in combination with clavulanic acid, for patients without a penicillin allergy. However, clindamycin or metronidazole is used in patients with a true penicillin allergy [2]. The combined therapy of amoxicillin and metronidazole is also recommended in some clinical situations, especially when amoxicillin/clavulanic acid, which offers anaerobic coverage, is unavailable. It should be noted, however, that while amoxicillin alone is the first line of therapy in odontogenic infections, there are fewer indications for the use of amoxicillin/clavulanic acid (or the combination of amoxicillin with metronidazole), such as infections that spread to the maxillary sinus. Therefore, the judicious use of amoxicillin/clavulanic acid is recommended to reduce its current overprescribing in dental practice [22]. Table 1 lists the most common indications for antibiotic therapy in dental practice, their bacterial coverage, and potential alternatives. Nevertheless, certain clinical circumstances may require the dentist’s judgment on a case-by-case basis, such as in patients with complex medical conditions, comorbidities, or allergies.

### 3.1. Endodontic Infections

In endodontic infections, adjuvant measures might be required in addition to standard endodontic treatment when an abscess forms. In such cases, achieving drainage should be the primary objective. When there is localized fluctuant swelling, drainage is considered adequate and does not require systemic antimicrobial therapy [25,31]. Antibiotics are indicated only in certain situations, namely in immunocompromised patients, when there is systemic involvement, in cases with a rapid onset of swelling, spreading cellulitis, when immediate treatment is not feasible, and in cases of persistent infections that do not respond to conventional root canal therapy or intracanal medications [25,27,31]. In these specific cases, the use of antibiotics can offer significant benefits, providing hope for improved patient outcomes.

### 3.2. Periodontal Infections

In chronic periodontitis patients, antibiotics might be recommended as an adjunct to non-surgical therapy. Systemic antibiotics, particularly the combination of amoxicillin and metronidazole, when used in addition to scaling and root planning (SRP), have been shown to result in a greater and statistically significant reduction in probing depth, a higher percentage of closed pockets, greater clinical attachment gain, and decreased bleeding upon probing [23,32,33].

Although systematic reviews have demonstrated additional benefits of antibiotic use [33,34,35], clinical practice guidelines developed by the European Federation of Periodontology do not recommend the routine use of antibiotics as an adjunct to SRP in periodontitis patients [30].Considering the global concerns regarding antibiotic use and the rise of antibiotic resistance, antibiotics should be considered only for specific patients, as listed below:

1. Young patients with generalized Stage III/IV periodontitis [30];
2. Patients with rapid disease progression or uncontrolled diabetes (i.e., grade C patients) [30];
3. Patients with necrotizing periodontitis or a periodontal abscess when there is clinical evidence of systemic involvement (a fever, lymphadenopathy, or malaise) [36,37]

### 3.3. Prevention of Postsurgical Infections in Immunocompetent Patients

For healthy (i.e., immunocompetent) patients, antibiotics are recommended for certain dental procedures to prevent postoperative infections and improve procedural outcomes. Such procedures include, for example, the removal of impacted third molars (if complications are anticipated or in case of deep bony impaction), dental implant placement, and procedures involving the placement of biomaterials [38].

#### 3.3.1. Third Molar Removal

The prescription of antibiotics following third molar removal is common to overcome postoperative infections, such as alveolitis and secondary infection, but it remains controversial. Due to the harmful side effects of antibiotics, more clinical trials are needed to find alternative methods that can aid in patient recovery during the postoperative period [39]. The most commonly prescribed antibiotic is amoxicillin/clavulanic acid [40].

Completely bone-impacted lower third molars are among the most challenging surgical extractions. Some surgeons prescribe prophylactic antibiotics one hour pre-operatively, as it has been shown to be more effective than a placebo in relieving pain, reducing edema, and improving mouth opening. However, it is not considered necessary to prevent postoperative infections; thus, postoperative antibiotic treatment can only be recommended for three days [40].

Delayed-onset infection is a rare postoperative complication, occurring in only 1.5% of cases after seven days postsurgery. Of these patients, only one-third responded well to antibiotics, with clindamycin being the first choice, followed by metronidazole and amoxicillin/clavulanate. The most common isolated bacterial strains were *Fusobacterium* spp., *Prevotella* spp., and *Peptostreptococcus*, which responded well to clindamycin, followed by metronidazole and amoxicillin/clavulanic acid [40].

#### 3.3.2. Dental Implants and Implant-Related Procedures

A Cochrane review concluded that antibiotics generally help reduce dental implant failure rates. The oral administration of 2 g of amoxicillin one hour before surgery may lower the risk of dental implant failure; however, the effectiveness of postoperative antibiotics remains debatable [24]. For sinus lift procedures, the lack of evidence makes it difficult to strongly support the use of antibiotics. Nonetheless, based on expert opinion, antibiotics are usually prescribed and can be started before surgery and continue for seven days postoperatively. Antibiotics are recommended in case of surgical complications, such as Schneiderian membrane perforation [28,29,41]. Guided bone regeneration with or without simultaneous implant placement is a common procedure in which antibiotics are usually prescribed. Unfortunately, few studies have investigated the additional benefit of antibiotics in preventing postoperative infections. Therefore, it should be emphasized that antibiotic use is recommended based on expert opinions until further studies provide strong evidence [26,28].

## 4. Indications for Prophylactic Antibiotics

Antibiotic prophylaxis is given to certain patients to prevent postoperative local or disseminated infections. Two main categories of patients are considered candidates for prophylactic antibiotics: patients at risk of IE and those at risk of infections due to their immunocompromised status.

### 4.1. Infective Endocarditis

Infective endocarditis is a serious bacterial infection that results in the inflammation of the inner lining of the heart and heart valves. It can lead to complications such as embolic events and heart failure [42]. Viridans group *Streptococci* from the oral flora are commonly implicated in this infection [43]. Therefore, prophylactic antibiotics are recommended to prevent IE in high-risk patients undergoing invasive dental procedures [43]. Cardiac conditions that put patients at high risk of IE include the following [43,44,45]:

1. Prosthetic heart valve or material:
  - Presence of prosthetic cardiac valve;
  - Transcatheter implantation of prosthetic valves;
  - Repair of a cardiac valve with devices, such as rings, clips, or annuloplasty;
  - Implantable heart or left ventricular assist devices.
2. Prior history of IE, relapse, or recurrence.
3. Cyanotic heart disease (CHD):
  - Unrepaired congenital CHD, including conduits and palliative shunts;
  - Fully repaired congenital heart defect with a prosthetic device or material, whether placed surgically or via a transcatheter, during the first six months after the procedure;
  - Repaired CHD with residual defects at or near the site of a prosthetic patch or device;
  - Surgical or transcatheter conduit placement or a pulmonary artery valve, such as the Contegra conduit or Melody valve.
4. Heart transplant recipients who develop cardiac valvopathy.

Dental procedures that necessitate the administration of prophylactic antibiotics to patients at high risk of IE include all procedures that involve the manipulation of the gingival tissue, the periapical region of the teeth, or oral mucosa perforation, such as periodontal/implant surgery, tooth extraction, root canal treatment, and scaling and root planning. The recommended antibiotics for IE prophylaxis are shown in Table 2, as recommended by the American Heart Association and the European Society of Cardiology [44,46].

### 4.2. Immunocompromised Patients

The immune system is essential for fighting infections, alongside appropriate antimicrobial therapy. Therefore, immunosuppression (i.e., a weakened immune system) can put a patient at risk of infections that would not otherwise impact immunocompetent individuals. Antibiotic prophylaxis should be considered in immunocompromised patients, and it is crucial to coordinate this with the physician managing the underlying immunocompromising condition to ensure safe dental care. The choice of prophylactic regimens may vary depending on the severity of the immunosuppression, such as the level of neutropenia and duration of immunosuppressive therapy. For example, the use of fluoroquinolones is preferred over other classes of antibiotics in cases of profound neutropenia (defined as <100 neutrophils/µL for >7 days) [48]. Therefore, dentists and physicians should collaboratively select the appropriate agent and the duration based on the patient’s condition.

Conditions that place patients at high risk of infections and therefore should be considered for prophylactic antibiotics include the following [27,49,50,51]:

1. Uncontrolled human immunodeficiency virus (HIV) infection;
2. Severe combined immunodeficiency (SCID);
3. Conditions associated with neutropenia, such as severe congenital neutropenia, patients undergoing cancer chemotherapy, immunosuppressive therapy for organ transplant or autoimmune disorders, or patients undergoing radiation therapy;
4. Hematopoietic stem cell or solid organ transplants;
5. Asplenia (post splenectomy);
6. Chronic high-dose steroid therapy (≥2 weeks of daily 20 mg or 2 mg/kg of prednisone or equivalent) [52];
7. Poorly controlled diabetes mellitus;
8. Renal hemodialysis.

## 5. Situations Wherein Antibiotics Are Being Overused in Dentistry

The overuse of antibiotics can result in unfavorable outcomes, such as the development of bacterial resistance and unnecessary exposure to adverse effects. In dental practice, there are several situations in which antibiotics are being overused without rational justification. Table 3 summarizes some of these common situations and the recommended antibiotic-sparing management actions.

### 5.1. Tooth Extraction

Antibiotics are often unnecessary for all types of teeth extraction. For instance, dentists may mistake signs of surgical trauma (e.g., postsurgical pain, swelling, and trismus) for postsurgical infections. These conditions should be managed with anti-inflammatory and antiseptic therapies rather than antibiotics [53,54]. Additionally, antibiotics have no role in the treatment of alveolar osteitis (dry socket), as it is not an infection but rather a complication of tooth extraction that is defined as an area of exposed bone with the absence of a blood clot, leading to the delayed healing of the extraction site after exodontia. Instead of antibiotics, treatment may involve using chlorhexidine mouthwash or performing a saline irrigation of the site, along with pain-relieving medications and patient reassurance [53,54].

### 5.2. Endodontic Infections Without Systemic Involvement

In immunocompetent patients, most endodontic infections without systemic involvement (i.e., irreversible pulpitis, pulp necrosis, acute apical periodontitis, chronic apical abscesses, or acute apical abscesses) do not require antibiotic therapy. Successful resolution can be achieved through root canal therapy, drainage in cases of localized fluctuant swelling, and pain medication in symptomatic cases. Therefore, the use of systemic antibiotics is generally unnecessary [25,27,31]. It is inappropriate to prescribe systemic antibiotics when there is a lack of blood supply to the infected site, as in the case of pulp necrosis, because the antibiotic would not effectively reach the infected area [4,57,58].

Furthermore, prescribing antibiotics to manage dental pain following an endodontic procedure is also inappropriate, as the pain results from an inflammatory process rather than an infectious process [25]. Pain can be effectively managed with opioid analgesics, such as hydrocodone or oxycodone, as well as nonsteroidal anti-inflammatory drugs (NSAIDs), such as ibuprofen or diclofenac [25].

### 5.3. Prophylaxis Against IE in Cardiac Patients Not at High Risk Undergoing Invasive Dental Procedures

Cardiac patients not at high risk for IE do not require antibiotic prophylaxis for invasive dental procedures that involve the manipulation of the gingiva, mucosa, or periapical region of the teeth; therefore, it should not be prescribed. Except for the conditions listed in Section 4.1 of this review, antibiotic prophylaxis is not recommended for patients with any other cardiac condition.

### 5.4. Prophylaxis for IE in High-Risk Cardiac Patients Undergoing Noninvasive Dental Procedures

Patients at high risk of IE who are undergoing procedures that do not involve the gingival tissue or periapical area, such as anesthetic injections through noninfected tissue, taking dental radiographs, the placement of removable appliances, the shedding of primary teeth, and bleeding from trauma to the lips or oral mucosa, do not need antibiotic prophylaxis for IE [44].

### 5.5. Prophylaxis for Prosthetic Joint Infections

Prescribing prophylactic antibiotics to prevent prosthetic joint infections is not recommended for all patients with prosthetic joints. Physicians should only consider prophylaxis in clinical situations in which there is a significant medical risk to the patient during an invasive dental treatment, such as treating a patient with a history of complications related to a prosthesis. In the presence of risk factors, prophylaxis is recommended after consulting the patient’s orthopedic physician [56].

## 6. Becoming an Antibiotic Steward

Antibiotic stewardship is defined by the Division of Oral Health of the US Centers for Disease Control and Prevention (CDC) as “the effort to measure antibiotic prescribing; to improve antibiotic prescribing by clinicians and use by patients so that antibiotics are only prescribed and used when needed; to minimize misdiagnosis or delayed diagnoses leading to underuse of antibiotics; and to ensure that the right drug, dose, and duration are selected when an antibiotic is needed” [59]. To support antibiotic stewardship in dental practice, the European Society of Endodontology recommends in their statement on antibiotic use in endodontics that antibiotics should be prescribed for 3 days while following up with the patient, and further antibiotics should be prescribed only if indicated clinically [31].

In the US, 10% of antibiotic prescriptions are written by dentists, and most are for outpatient use [60]. Antibiotics are the most frequently prescribed medications in dentistry, with β-lactams at the top of the list. A study in Australia between 2006 and 2018 found that 11 of the top 20 medications prescribed in dentistry were antibiotics. An additional study revealed significant increases (about 50–400%) in the prescription of opioids, benzodiazepines, and antibiotics (most frequently amoxicillin and metronidazole) by Australian dentists between 2002 and 2017 [61]. A further study found that antibiotic prophylaxis was unnecessary in most pre-dental visits (over 80%) and that the guidelines were not followed in 58–81% of dental antibiotic prescriptions, especially for infection prevention [62].

Stewardship initiatives are crucial in reducing the problems caused by antibiotic misuse, notably bacterial resistance, microbiota divergence, and digestive and hematological issues. In fact, antibiotic resistance is a serious worldwide issue that leads to increased morbidity, mortality, and healthcare costs [63]. Therefore, antibiotic prescribing should be improved in dental practices, which could be accomplished by implementing antimicrobial stewardship initiatives [64,65]. Some studies have shown the successful implementation of antibiotic stewardship interventions [60,66].

Below are recommendations for dentists to practice antibiotic stewardship, even in the absence of a formal stewardship program at their institution [3,4,59,60,64,67]:

1. Make a commitment to optimize antibiotic use;
2. Create an improved prescription policy for dentists, monitor antibiotic use and associated outcomes, and provide feedback to practitioners;
3. Enable access to the knowledge required to enhance antibiotic prescriptions (i.e., provide access to reliable drug references);
4. Provide education and training for dentists in different specialties and collaborate with pharmacists (especially those specialized in infectious diseases) to provide education pertinent to appropriate antibiotic use;
5. Only acute infections should be treated with narrow-spectrum antibiotics;
6. Only patients at a high risk of complications following dental procedures should receive antibiotic prophylaxis;
7. Advise patients on proper dental care, hygiene, and the use of oral healthcare products;
8. When antibiotics are necessary, ensure the appropriate medication, dosage, and duration are prescribed (Table 1).

## 7. Collaborative Work Between Dentists and Pharmacists

**Figure 2:**
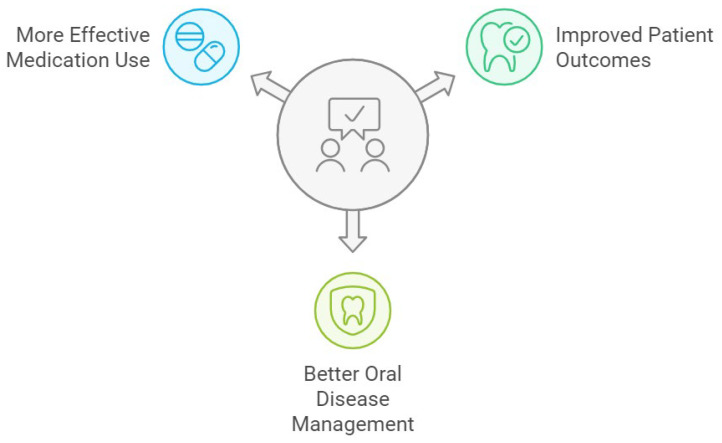
Outcomes of collaboration between dentists and pharmacists.

Interprofessional collaboration between dentists and other healthcare professionals can help achieve better patient care [68]. One such example is the collaboration between dentists and pharmacists, which can involve providing medication therapy consultations, reviews of prescription orders, and patient counseling regarding the appropriate use of medications [69]. Such a collaboration would result in favorable outcomes pertinent to patient care and the prevention of the emergence of antimicrobial resistance when antibiotics are appropriately prescribed (Figure 2) [70]. One study by Johnson et al. investigated the outcomes of the collaboration of pharmacists (including pharmacy trainees) with dental college students and faculty [71]. In that collaborative project, the pharmacy team provided medication therapy management, including medication reconciliation, counseling, identifying drug-related problems, and making recommendations for prescribed medications. Of the 6596 patients reviewed, the pharmacy team made 2773 interventions for 2438 patients. The interventions included identifying adverse reactions or drug interactions, drug choice problems, and dosing problems. The study highlights that the involvement of pharmacists in dental practice resulted in improved patient care.

Table 4 lists some potential strategies that can be used by dentists and pharmacists to ensure better oral disease management and patient care.

## 8. Conclusions

Antibiotic prescribing in dental practice should be limited to cases with documented infections or as prophylaxis for high-risk patients. Every dentist can practice antibiotic stewardship and become an antibiotic steward by prudently prescribing antibiotics only when necessary. To accomplish this, dentists should have the knowledge to identify such cases and exclude those who do not meet the criteria for antibiotic use. Collaboration with drug experts, such as pharmacists (particularly those specializing in infectious diseases), is encouraged to provide such recommendations and perhaps implement antibiotic stewardship interventions, such as developing antibiotic prescription protocols. Lastly, the tables provided in this review can be used as a quick guide for dentists when the indication for antibiotics is questionable.

## Figures and Tables

**Table 1:** Antibiotics indicated for treatment in dental practice [23,24,25,26,27,28,29,30,31].

Antibiotic (Route of Administration and Dose)	Bacterial Coverage	Indication for Treatment or Prophylaxis	Safety Considerations	Alternatives
β-lactams				
Amoxicillin (PO): 500 mg q8h Ampicillin (IV): 50–100 mg/kg (up to 2 g) q6h	G+ (all *Streptococci, Staphylococci* *, *Enterococcus faecalis*) Some G− (*Escherichia coli*, *Klebsiella* spp., *Proteus* spp.)	Acute apical abscess (localized fluctuant swelling) in immunocompromised patients (3–7 days) ** Acute apical abscess (localized fluctuant swelling) when immediate treatment is not feasible (3–7 days) Acute apical abscess with systemic involvement (fever, malaise, trismus, lymphadenopathy) (3–7 days) Rapidly progressing infection (rapid onset of swelling in <24 h) (3–7 days) Cellulitis or facial space infection (3–7 days) Persistent infection that did not respond to root canal therapy and intracanal medications (3–7 days) In implant placement surgery o Preoperative single dose (amoxicillin 2 g, 1 h before surgery) o Continue postoperatively for 5–7 days in case of immediate implant Surgeries involving bone grafts, non-autogenous soft tissue grafts, or sinus lift. o Might start one day pre-operatively and continue for a total of 5–7 days	Safe during pregnancy, when breastfeeding, and for pediatrics	Non-IgE-mediated penicillin allergy (e.g., mild rash): o Cephalexin (PO): 250 mg q6h for 3–7 days o Cefazolin (IV or IM): 500 mg q8h for 3–7 days o Ceftriaxone (IV or IM): 1 g q24h for 3–7 days IgE-mediated penicillin allergy (e.g., anaphylaxis): o Azithromycin (PO or IV): 500 mg once, then 250 mg q24h for a total of 5 days o Clarithromycin (PO or IV): 500 mg once, then 250 mg q12h for 3–7 days o Clindamycin (PO or IV): 300 mg q6h for 3–7 days
Amoxicillin (PO): 250 mg q8h or 500 mg q12h + metronidazole (non β-lactam) (PO): 500 mg q8h Amoxicillin/clavulanic acid (PO): 1 g (875 mg amoxicillin/125 mg clavulanic acid) q12h	Same as above plus: Anaerobes (*Bacteroides* spp., *Prevotella* spp., *Clostridium* spp., *Peptostreptococcus* spp.)	Same indications as above in case of lack of response Young patients with generalized periodontitis stage III/IV or patients with rapid disease progression or uncontrolled diabetes, such as grade C patients. o As adjunctive to the non-surgical periodontal therapy to improve outcomes o The recommended regimen is amoxicillin + metronidazole (or amoxicillin/clavulanic acid) Necrotizing periodontitis with systemic involvement or in an immunocompromised patient ** Periodontal abscess in an immunocompromised patient or in case of a systemic involvement or		Same regimens as above (except clindamycin) plus metronidazole (PO or IV): 500 mg q6h for 3–7 days
**Macrolides**				
Erythromycin (PO): 250	Some G+ (all *Streptococci*, *Staphylococci* *) Some G− Atypical bacteria	2nd line for odontogenic infections	Safe during pregnancy, when breastfeeding, and for pediatrics	-
Azithromycin (PO or IV): 500 mg once, then 250 mg q24h for a total of 5 days				-
Clarithromycin (PO or IV): 500 mg once, then 250 mg q12h for 3–7 days		2nd line for periodontitis		-
**Tetracyclines**				
Doxycycline (PO): 100 mg q12h for 3–7 days Minocycline (PO): 200 mg once, then 100 mg q12h for 3–7 days	G+ (all *Streptococci*, *Staphylococci* *, *Enterococcus faecalis*) Some G− (*E. coli*, *Klebsiella* spp., *Enterobacter* spp., *Citrobacter* spp., *Serratia* spp., *Acinetobacter* spp.)	Replantation of avulsed teeth 2nd line for treatment of periodontitis as an adjunct to non-surgical therapy Not commonly suggested for the treatment of odontogenic infection	Avoid during pregnancy, while breastfeeding, and for pediatrics	Consider topical therapy
**Other antibiotics**				
Clindamycin (PO or IV): 600 mg once, then 300 mg q6h for 3–7 days	Some G+ (all *Streptococci*, *Staphylococci*, including MRSA) Anaerobes	Patients with persistent infections (excellent choice for patients with allergy to β-lactams)	Avoid during pregnancy, when breastfeeding, and for pediatrics	-
Metronidazole (PO or IV): 500 mg q6h for 3–7 days	Anaerobes	For patients with periodontal and odontogenic infections; administered in combination with amoxicillin or ampicillin for the conditions mentioned above		-

* Excluding methicillin-resistant *Staphylococcus aureus* (MRSA). ** Please refer to Section 4.2 (immunocompromised patients) of this review for a detailed list of conditions meeting this definition. G+, Gram-positive; G−, Gram-negative; IM, intramuscular; IV, intravenous; PO, oral.

**Table 2:** Antibiotics indicated for infective endocarditis prophylaxis in high-risk patients undergoing invasive dental procedures to be given as a single dose 30–60 min before the procedure [44,46].

Route of Administration	Allergy Status	Agent	Dose
Oral	No allergy	Amoxicillin	2 g
Oral	Allergic to penicillins *	Cephalexin	2 g
Oral	Allergic to all β-lactams	Azithromycin	500 mg
Oral	Allergic to all β-lactams	Clarithromycin	500 mg
IM or IV	No allergy	Ampicillin	2 g
IM or IV	Allergic to penicillins *	Cefazolin	1 g
IM or IV	Allergic to penicillins *	Ceftriaxone	1 g

Note: Clindamycin is no longer recommended as per the latest guidelines. The same antibiotics are recommended in pediatrics, though different doses that are weight-based are used. * Non-IgE-mediated allergy, such as mild rash. IgE-mediated allergies include anaphylaxis, angioedema, and urticaria [47].

**Table 3:** Cases in which antibiotics are not indicated and the recommended antibiotic-sparing management actions.

Case	Recommended Management Actions
1. All types of tooth extraction [53,54]	Antiphlogistic and antiseptics
2. Dry socket [53,54]	Chlorhexidine mouthwash or saline site irrigation and analgesics
3. Pain of endodontic origin without signs and symptoms of systemic infection (symptomatic irreversible pulpitis and symptomatic apical periodontitis) [25,27]	Root canal therapy
4. Teeth with necrotic pulps and a radiolucency [25]	Root canal therapy, or extraction if the tooth is non-restorable
5. Teeth with a sinus tract (chronic apical abscess) [25]	Root canal therapy, or extraction if the tooth is non-restorable
6. Acute apical abscess in immunocompetent patients (localized fluctuant swelling) when same-visit treatment is feasible [25,27]	Root canal therapy with incision and drainage, or extraction if the tooth is non-restorable
7. Dental pain of endodontic origin or after an endodontic procedure [25,55]	Opioid analgesics or non-steroidal anti-inflammatory drugs
8. Prophylaxis for IE in cardiac patients not at high risk undergoing invasive procedures [44]	Prophylaxis is not indicated (Section 5.3)
9. Prophylaxis against IE in high-risk patients undergoing noninvasive procedures [44]	Prophylaxis is not indicated
10. Prophylaxis to prevent prosthetic joint infection in patients without risk factors [56]	Prophylaxis is not indicated

**Table 4:** Strategies for effective collaborations between dentists and pharmacists.

Strategy	Explanation
1. Medication therapy consultations	By keeping open lines of communication between dentists and pharmacists, both professionals could discuss patient information and agree on appropriate treatment plans.
2. Treatment protocol development	Treatment protocols for major dental conditions encountered by specialized dentists could be prepared in collaboration with pharmacists.
3. Interdisciplinary meetings	Organizing regular meetings between dental and pharmacy teams can help identify areas for collaboration, discuss patient cases, and develop joint strategies for patient care.
4. Continuing education	Promoting joint continuing education dental–pharmacy programs can keep both professions updated on the latest advancements in oral health and pharmacotherapy, enhancing their mutual understanding of their respective roles.
